# Supplementary material for: Aβ-Induced Damage Memory in hCMEC/D3 Cells Mediated by Sirtuin-1
Source: Int J Mol Sci. 2020 Nov 3;21(21):8226. doi: 10.3390/ijms21218226 (PMC7662699; doi:10.3390/ijms21218226)
Supplement: Supplementary file 1 [file ijms-21-08226-s001.pdf]

Supplementary data

Aβ-induced damage memory in hCMEC/D3 cell mediated by sirtuin-1

Authors: HaoChen Liu, Yixuan Zhang, Hong Zhang, Sheng Xu, Huimin Zhao and XiaoQuan Liu,*

Center of Drug Metabolism and Pharmacokinetics, China Pharmaceutical University, Nanjing, 210009, China
E-Mail address: haochenliu@cpu.edu.cn (HCL); yxzhang@stu.cpu.edu.cn (YXZ); 296481679@qq.com (HZ); 570302057@qq.com (SX); 1060046870@qq.com (HMZ) lxq@cpu.edu.cn (XQL, Corresponds author).
* Corresponding author: lxq@cpu.edu.cn (XQL, Corresponds author). Tel.: + 86-25-83271260

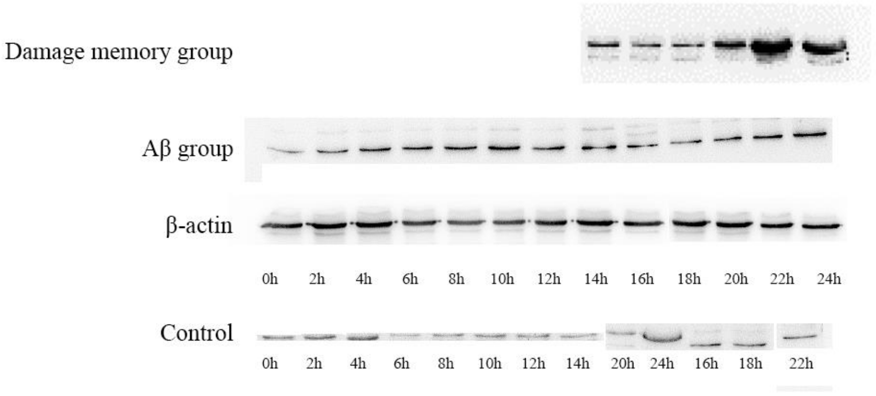

Figure S1. the time course of *p66*SHC levels in control group, $A\beta$ group and damage memory group.

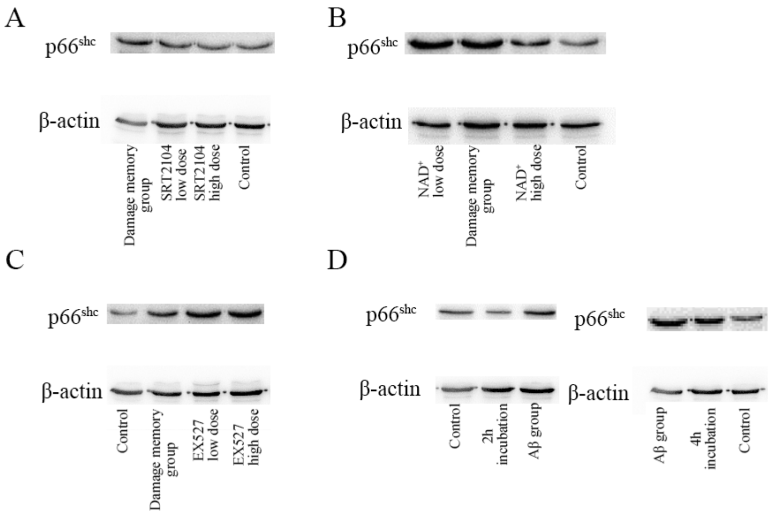

Figure S2. A: the change of *p66*^{SHC} in SRT2104 treated hCMEC/D3 cell. B: the change of *p66*^{SHC} in NAD^+ supplement treated hCMEC/D3 cell. C: the change of *p66*^{SHC} in EX527 treated hCMEC/D3 cell. D: the results of *p66*^{SHC} level in the experimental validation of cerebrovascular endothelial cell damage memory formation time.

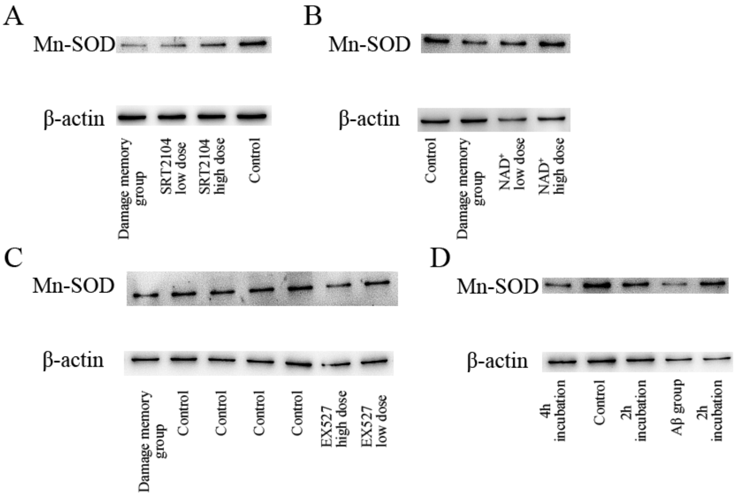

Figure S3. A: the change of Mn-SOD in SRT2104 treated hCMEC/D3 cell. B: the change of Mn-SOD in NAD^+ supplement treated hCMEC/D3 cell. C: the change of Mn-SOD in EX527 treated hCMEC/D3 cell. D: the results of Mn-SOD level in the experimental validation of cerebrovascular endothelial cell damage memory formation time.

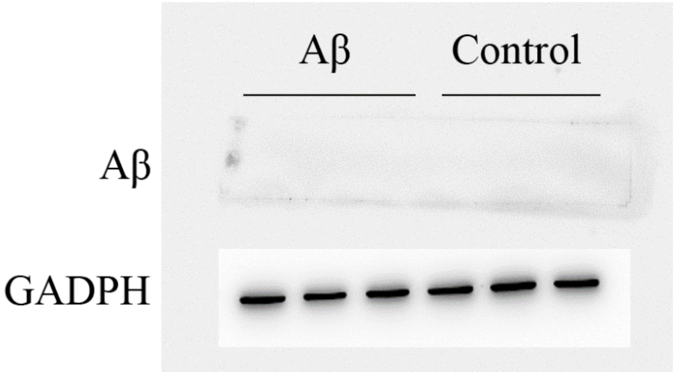

Figure S4. the intracellular Aβ accumulation in Aβ group and control group.

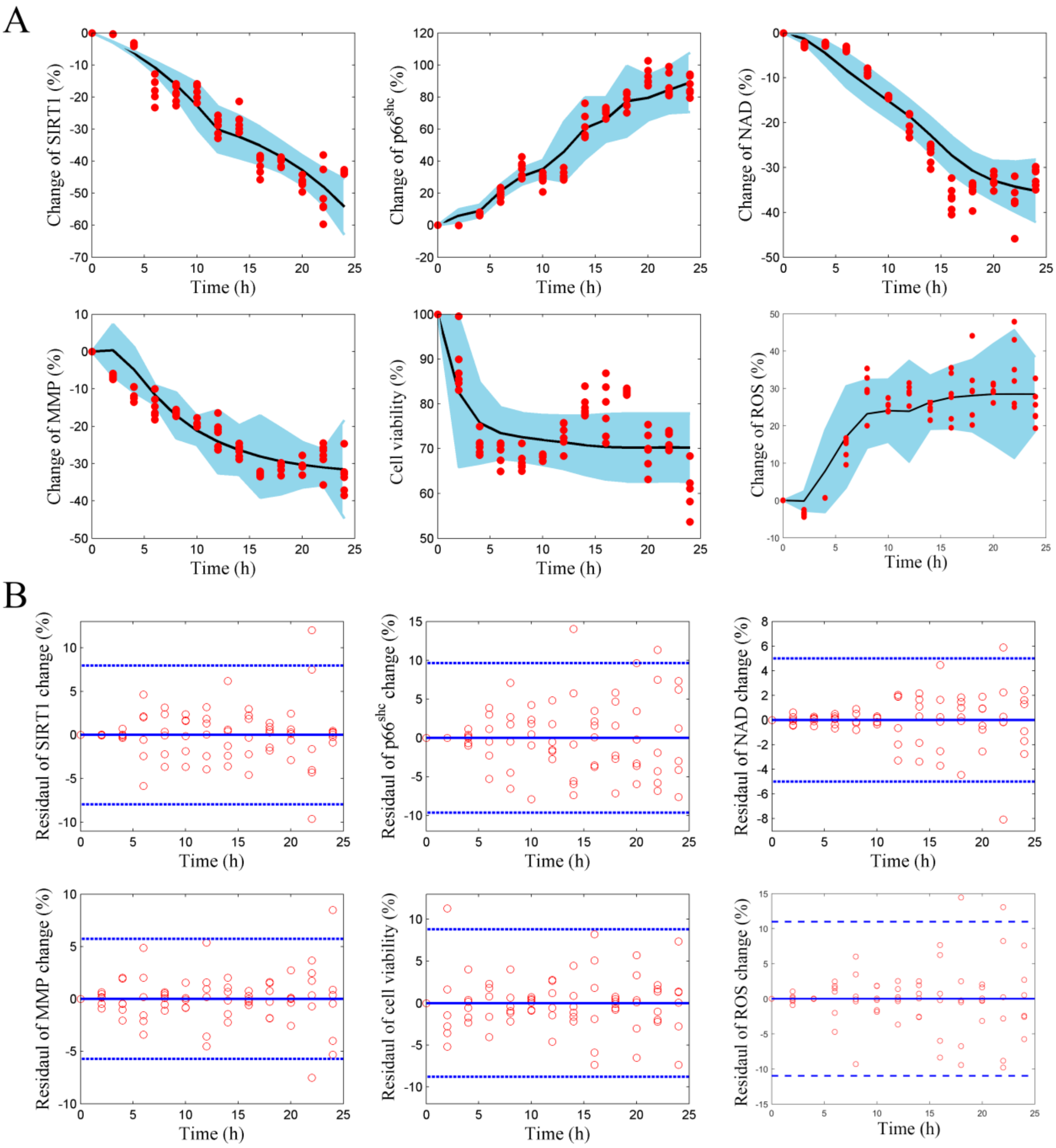

Figure S5. A: visual predicted check (VPC) for sirt-1, $p66^{SHC}$, ROS, NAD^{+}, MMP, and cell vitality. The shaded area represents the 95% confidence interval of the simulated median value. The line represents the median value of observed value. The red scatters represent observed values. B: scatter plots of predicted residuals vs. time for sirt-1, $p66^{SHC}$, ROS, NAD^{+}, MMP, and cell vitality.

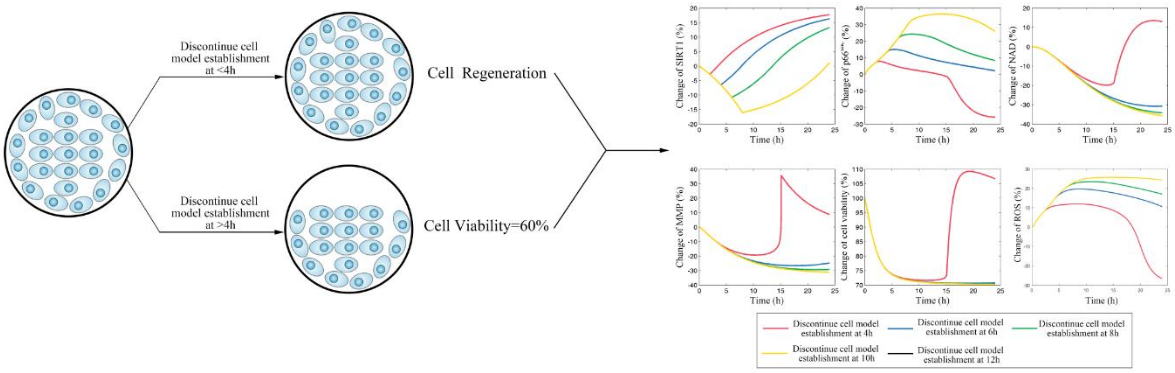

Figure S6. the simulation results for scenario I which investigate the time of cerebrovascular endothelial cell damage memory formation.

Table S1. mechanism based kinetic process model parameters and bootstrap validation.

Parameters	Estimate	CV	CI		Bootstrap
			LL	UL	
k_{in}^{SIRT1}	0.02	77.05%	4.73E-03	0.04	0.02
$E_{A\beta}$	-0.63	77.05%	-1.12	-0.15	-0.63
k_{out}^{SIRT1}	0.10	35.96%	0.07	0.14	0.11
$k_{in}^{p66^{SHC}}$	0.06	65.20%	0.02	0.11	0.06
$k_{p66^{SHC}}^{SIRT1}$	28.29	67.40%	9.22	47.36	28.29
$k_{p66^{SHC}}^{NAD^+}$	13.05	81.77%	2.38	23.73	13.50
$k_{out}^{p66^{SHC}}$	1.42	7.29%	1.31	1.52	1.42
k_{in}^{ROS}	1.11	0.22%	1.10	1.11	1.11
$E_{\max}^{p66^{SHC}}$	0.32	19.86%	0.26	0.38	0.35
$EC_{50}^{p66^{SHC}}$	0.11	96.27%	4.01E-03	0.21	0.11
$k_{out}^{p66^{SHC}}$	1.11	0.22%	1.10	1.11	1.11
$k_{in}^{NAD^+}$	0.26	58.87%	0.11	0.41	0.27
$k_{out}^{NAD^+}$	0.21	78.28%	0.05	0.38	0.23
k_{in}^{MMP}	0.86	88.70%	0.05	1.58	0.71
$E_{\max}^{ROS}$	1.11	0.15%	1.17	1.17	1.04
EC_{50}^{ROS}	0.03	0.11%	0.03	0.03	0.03
k_{out}^{MMP}	1.17	92.09%	0.03	2.18	1.02
k_{in}^{MTT}	0.94	83.68%	0.15	1.72	0.94
$E_{\max}^{MMP}$	0.26	15.85%	0.21	0.30	0.26
EC_{50}^{MMP}	0.04	78.74%	0.01	0.08	0.04
k_{out}^{MTT}	1.04	78.53%	0.22	1.85	1.04
